# Whole Exome Sequencing and Segregation Analysis Confirms That a Mutation in *COL17A1* Is the Cause of Epithelial Recurrent Erosion Dystrophy in a Large Dominant Pedigree Previously Mapped to Chromosome 10q23-q24

**DOI:** 10.1371/journal.pone.0157418

**Published:** 2016-06-16

**Authors:** Benjamin R. Lin, Derek J. Le, Yabin Chen, Qiwei Wang, D. Doug Chung, Ricardo F. Frausto, Christopher Croasdale, Richard W. Yee, Fielding J. Hejtmancik, Anthony J. Aldave

**Affiliations:** 1 Stein Eye Institute, David Geffen School of Medicine at UCLA, Los Angeles, California, United States of America; 2 Ophthalmic Genetics and Visual Function Branch, National Eye Institute, National Institutes of Health, Bethesda, Maryland, United States of America; 3 Davis Duehr Dean Clinic, Madison, Wisconsin, United States of America; 4 Cross Ophthalmology Associates, Houston, Texas, United States of America; Cedars-Sinai Medical Center; UCLA School of Medicine, UNITED STATES

## Abstract

**Purpose:**

To report identification of a *COL17A1* mutation in a family with a corneal dystrophy previously mapped to chromosome 10q23-q24.

**Methods:**

Whole-exome sequencing was performed on DNA samples from five affected family members and two unrelated, unaffected individuals. Identified variants were filtered for those that were: located in the linked interval on chromosome 10q23-q24; novel or rare (minor allele frequency ≤0.01); heterozygous; present in all affected individuals and not in controls; and present in genes that encode proteins expressed in human corneal epithelial cells (reads per kilobase per million ≥1). Sanger sequencing of identified variants (SNVs) was performed in additional family members. *In silico* analysis was used to predict the functional impact of non-synonymous variants.

**Results:**

Three SNVs located in two genes were identified that met the filtering criteria: one rare synonymous c.3156C>T variant in the collagen, type XVII, alpha I (*COL17A1*) gene; and two rare variants, one synonymous and one missense, in the dynamin binding protein (*DNMBP*) gene. Sanger sequencing of additional family members determined that only the *COL17A1* variant segregates with the affected phenotype. *In silico* analysis predicts that the missense variant in *DNMBP* would be tolerated.

**Conclusions:**

The corneal dystrophy mapped to chromosome 10q23-q24 is associated with the c.3156C>T variant in *COL17A1*. As this variant has recently been identified in five other families with early onset recurrent corneal erosions, and has been shown *in vitro* to introduce a cryptic splice donor site, this dystrophy is likely caused by aberrant splicing of *COL17A1* and should be classified as epithelial recurrent erosion dystrophy.

## Introduction

The corneal dystrophies are a group of inherited disorders associated with bilateral, symmetric, and progressive loss of visual acuity due to the loss of corneal clarity. Reis-Bücklers corneal dystrophy (RBCD; MIM 608470) and Thiel-Behnke corneal dystrophy (TBCD; MIM 602082) are two forms of epithelial-stromal transforming growth factor beta induced (TGFBI) corneal dystrophies. RBCD and TBCD are associated with similar phenotypes, presenting with recurrent corneal erosions and the development of a “geographic” (RBCD) or “honeycomb” (TBCD) pattern of the anterior corneal layer.[1–3] These two dystrophies share a common genetic origin, with RBCD and TBCD associated with the p.(Arg124Leu) and p.(Arg555Gln) mutations in the transforming growth factor beta induced (*TGFBI*) gene on chromosome 5q31, respectively.[4–7]

A previous report by Yee et al. described a large pedigree affected with a dystrophy of the anterior corneal layer in which a *TGFBI* mutation was not identified.[8, 9] The majority of affected family members were described as demonstrating bilateral “honeycomb” opacities, and curly fibers were noted on electron microscopic examination of a corneal specimen from an affected individual, leading to the diagnosis of TBCD.[9] However, the resultant classification of this family as having TBCD has been questioned, primarily due to disagreement regarding whether the published photographs demonstrate a honeycomb-like pattern.[10, 11] In addition, the electron microscopic images of a corneal specimen from the proband’s sister were never published, and thus could not be independently evaluated. Linkage analysis mapped the dystrophy to an approximately 25 Mbp region on chromosome 10q23-q24 with a maximum multipoint LOD score of 5.5.[8] Examination of the genes mapped to the linked interval on chromosome 10q23-q24 led to the identification of *COL17A1* as both a positional and functional candidate, but screening of the gene did not reveal any presumed pathogenic variants.[12] Thus, both the clinical and genetic characterization of chromosome 10q23-q24 linked corneal dystrophy remained ambiguous.

Given the failure to identify the genetic basis of chromosome 10q23-q24 linked corneal dystrophy using a positional candidate gene approach, we decided to use whole exome sequencing (WES), focusing initially on variants identified in the previously linked interval. This led to the identification of three variants in two different genes that met all of the filtering criteria, with only one of the variants, c.3156C>T (p.(Gly1052 =)) in *COL17A1*, segregating with the affected phenotype in additional family members. Jonsson and colleagues recently reported the results of *in silico* modeling and an *in vitro* splice assay demonstrating that the c.3156C>T (p.(Gly1052 =)) synonymous variant in *COL17A1* leads to the introduction of a splice donor site, resulting in an aberrantly spliced transcript.[11] Even more recently, Oliver and colleagues independently identified the c.3156C>T variant in *COL17A1* segregating with the affected phenotype in three families with corneal dystrophies characterized by the early onset recurrent corneal erosions.[13, 14] Here, we report the use of whole exome sequencing (WES) to identify the genetic basis of what should be classified as epithelial recurrent erosion dystrophy in the original large pedigree that mapped the condition to chromosome 10q23-q24.

## Methods

This study followed the Declaration of Helsinki, adhered to the ARVO statement on human subjects, and was approved by the Institutional Review Board at the University of California at Los Angeles (UCLA IRB#11–000020). Informed written consent was obtained from all subjects in this study.

### Determination of affected status

The most recently published pedigree of the family with chromosome 10q23-q24 linked corneal dystrophy was used as a basis for determining the affected status of family members.[9] For previously examined family members who were subsequently reexamined, and for previously unexamined family members, determination of the affected status was based on a history of recurrent corneal erosions and the presence of subepithelial opacification, likely representing scarring in the location of a prior corneal erosion.

### DNA collection

**Fig 1:**
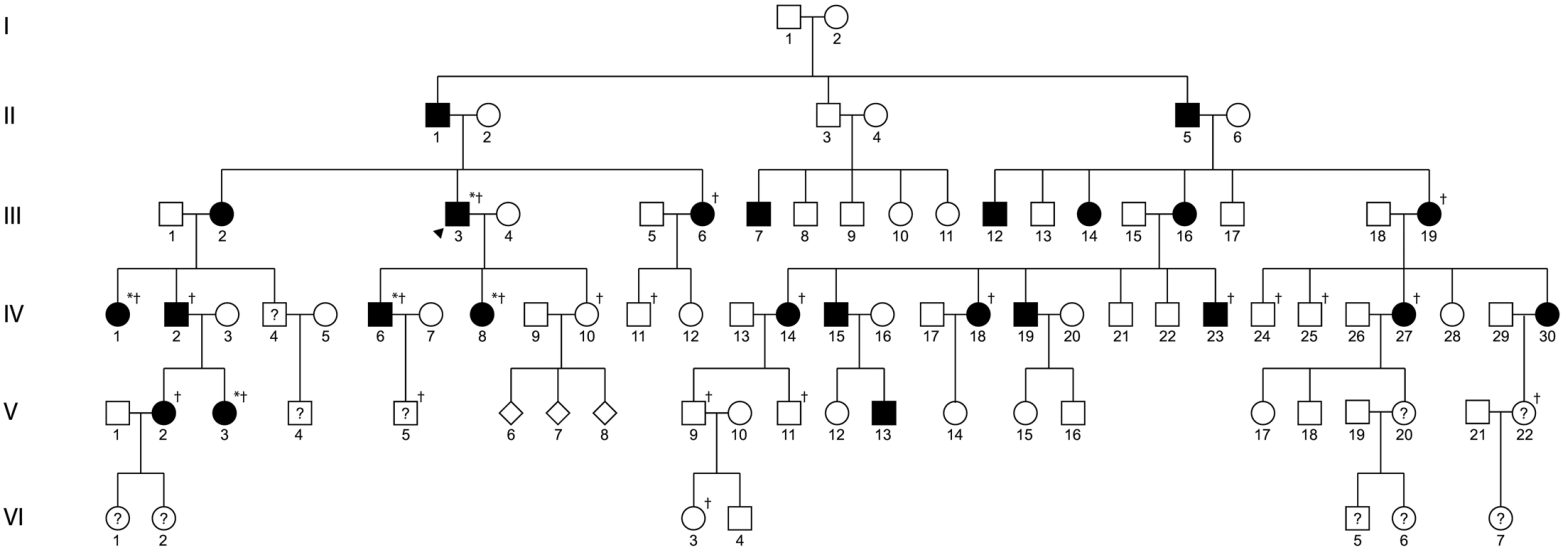
Pedigree of a family with chromosome 10q23-q24 linked dystrophy. Females are represented by circles, males by squares, and individuals of unknown gender by diamonds. Affected individuals are shown with filled symbols and unaffected individuals are shown with open symbols. Individuals of unknown affected status are shown with a question mark. The arrow head indicates the proband. An asterisk (*) indicates individuals that underwent WES. A dagger (†) indicates individuals that underwent Sanger sequencing for identified candidate variants in *COL17A1* and *DNMBP*.

DNA samples were collected for WES from five affected family members (Fig 1: III-3, IV-1, IV-6, IV-8, V-3). Previously collected DNA samples were used for Sanger sequencing of potentially pathogenic variants identified with WES.

### WES and variant calling

WES was performed using genomic DNA samples from the aforementioned five affected individuals and two unrelated, unaffected controls at the UCLA Clinical Microarray Core. DNA libraries were prepared using the TruSeq DNA Sample Preparation Kit v2 (Illumina, Inc., San Diego, CA) and exome capture was performed with the SeqCap EZ Exome Library v3.0 (Roche NimbleGen, Inc., Madison, WI). Paired-end sequencing (2x100 bp) was performed with Illumina’s HiSeq 2500. Using the Partek Flow platform (Partek Inc., St. Louis, MO), the resulting reads were aligned to the human hg19 reference genome with Bowtie2, and variant identification was performed with SAMtools mpileup.

#### WES data analysis and filtering

Using Partek Genomics Suite, multiple criteria were used to filter detected single nucleotide variants (SNVs) to identify those that were: located in a region of ≥ 5X read depth and ≥ 20 base quality score; located in the linked interval on chromosome 10q23-q24; novel or rare (minor allele frequency ≤ 0.01 per The Database of Single Nucleotide Polymorphisms [dbSNP Build ID 138], National Center for Biotechnology Information, National Library of Medicine, Bethesda, MD); heterozygous, identified by allele frequency of 0.5; present in all five affected individuals; absent in both unrelated, unaffected controls; and expressed in *ex vivo* human corneal epithelial cells (reads per kilobase per million ≥1) (Frausto and Aldave, unpublished data recorded in 2014). Quality score values were calculated by Partek Genomics Suite using default settings. Filtered variants were then annotated with RefSeq database compiled on May 7, 2015. While dbSNP was used to determine the minor allele frequency of previously identified variants and perform the filtering for novel or rare variants, the NHLBI Exome Sequencing Project (ESP) (Seattle, WA), EVS data release ESP6500SI-V2, was used to validate the minor allele frequency of filtered variants.

### Polymerase chain reaction (PCR) amplification and automated sequencing

DNA samples from 13 affected family members, seven unaffected family members and two family members of undetermined affected status were screened for sequence variants that met each of the aforementioned filtering criteria using custom designed PCR primers (Table 1). PCR was performed in 10μL reaction volumes containing 40 ng of genomic DNA, 2.5 picomoles of each primer, 1 μL GeneAmp 10×PCR Gold buffer (Applied Biosystems, Grand Island, NY), 0.8 μL 10 mM dNTP mix (Applied Biosystems, Grand Island, NY), 0.6 μl 25 mM MgCl_2,_ and 1 μL Taq DNA polymerase. Touchdown PCR amplification consisted of a denaturizing step at 95°C for 5 min, followed by decreasing the annealing temperature from an initial 64°C in a stepwise fashion by 0.5°C every second cycle for 15 cycles, then an annealing temperature of 57°C for 20 cycles and finally a prolonged elongation step at 72°C for 10 min. The PCR primers were used for bidirectional sequencing using the BigDye Terminator Ready reaction mix according to the manufacturer’s instructions (Applied Biosystems, Grand Island, NY). Sequencing was performed on an ABI PRISM 3130 Automated Sequencer (Applied Biosystems, Grand Island, NY).

**Table 1:** Filtered coding region variants in positional candidate genes for the corneal dystrophy linked to chromosome 10q23-q24.

Gene	Genomic Position	Protein Change	Transcript	Reference	Alternative	RefSeq number	Minor allele frequency
*COL17A1*^a^	g.105,797,446	p.(Gly1052 =)	NM_000494	G	A	None	N/A
*DNMBP*^b^	g.101,667,814	p.(Met831Thr)	NM_015221	A	G	rs17854134	0.0088^c^ 0.0235^d^
*DNMBP*^b^	g.101,667,792	p.(Ser2606 =)	NM_015221	C	T	rs17854135	0.0096^c^ 0.0258^d^

^a^Sequenced with (5’ to 3’): forward primer CTTTGTTCCTTGGTCGGCAG; reverse primer CAGCAAACGAGGAGATGAGG

^b^Sequenced with (5’ to 3’): forward primer ATCTTCCCGGAGGCATAGTT; reverse primer GCTCATTTCCGTGCATTTTT

^c^Minor allele frequency from The Database of Single Nucleotide Polymorphisms [dbSNP Build ID 138]

^d^Minor allele frequency from NHLBI Exome Sequencing Project (Seattle, WA), ESV data release ESP6500SI-V2

### *In silico* protein analysis

Polyphen-2, SIFT and PANTHER were used to predict the functional impact of segregating missense variants that met the filtering criteria.[15–17] In addition, splice site prediction programs, NetGene2 and MutPred Splice, were used to predict if synonymous substitutions created a cryptic splice donor site.[18, 19]

## Results

### Determination of affected status

**Fig 2:**
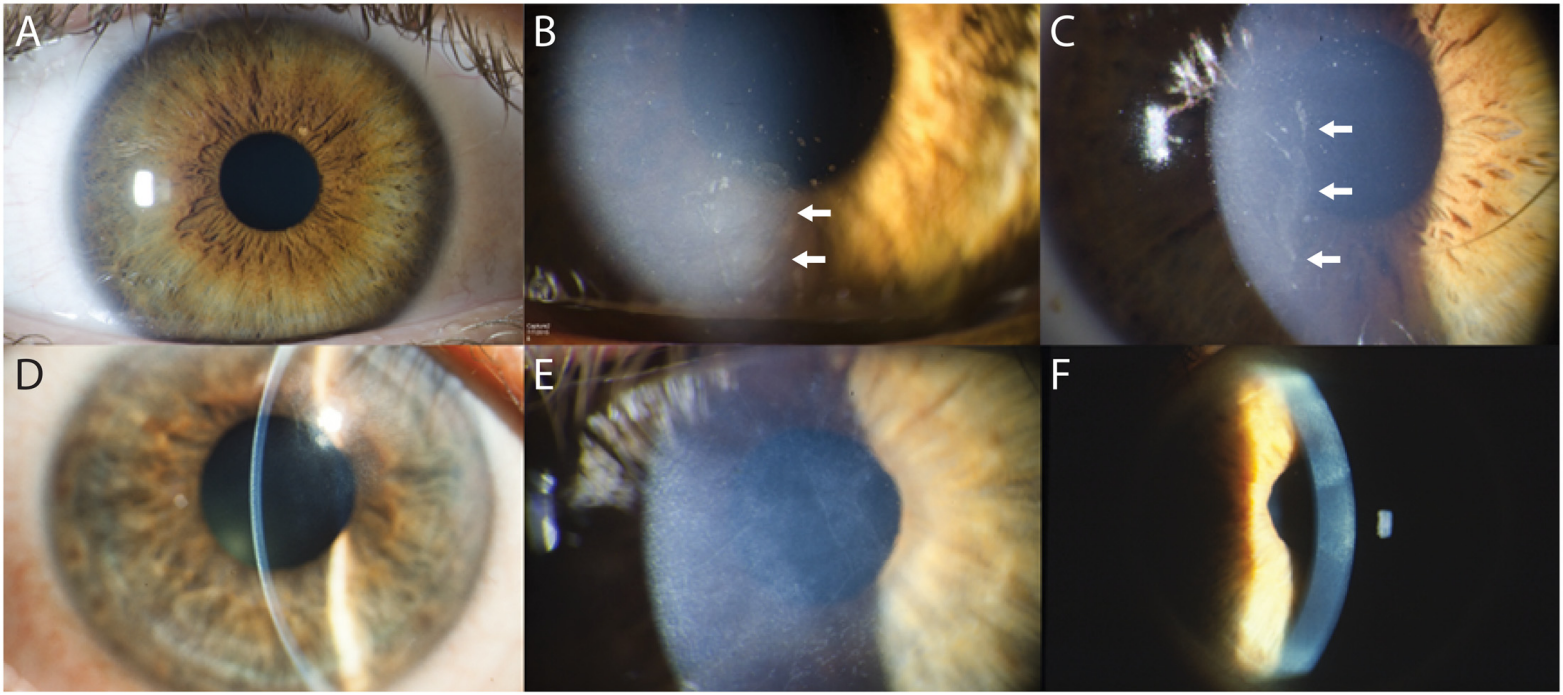
Clinical features of chromosome 10q23-q24 linked dystrophy. A. Affected individual V-2 (28 years old) demonstrates a clear cornea in the right eye. B. Three months later, she developed a corneal erosion (arrows). C. Affected individual V-3 (24 years old) also demonstrated clear corneas prior to the development of a corneal erosion, the location of which can be identified by the opacification of the corneal epithelium (arrows). D. Affected individual IV-1 (72 years old) demonstrates diffuse subepithelial opacification secondary to chronic recurrent corneal erosions seen with slit and diffuse illumination in the right (D and E) and left (F) eyes.

Examination of younger affected individuals revealed clear corneas, with the diagnosis made based on the development of spontaneous corneal erosions (Fig 2A–2C). Several decades after the onset of the recurrent corneal erosions, scattered or diffuse subepithelial opacification secondary to subepithelial scarring was observed (Fig 2D–2F). While such scarring did impair vision, it was also typically associated with a decrease in the frequency and severity of episodes of corneal erosion.

Two individuals (Fig 1: V-5 and V-22) were diagnosed as unaffected when initially examined at ages 20 (V-5) and 22 (V-22). Thus, both appear as unaffected in previously published pedigrees.[8, 12] However, when reexamined nine years later, both were subsequently reclassified as affected (Dr. Yee, unpublished communication). In the most recently published pedigree, individual V-22 is denoted as affected, although individual V-5 is still denoted as unaffected.[9] For individual V-22, the change in classification was made based on the presence of 3 focal corneal opacities, although no explanation was given for the change in affected status of individual V-5. Of note, this individual’s status remained unaffected in the most recently published pedigree (individual III-4).[9] Based on the uncertain affected status of both individuals V-5 and V-22, we have changed the status of both to undetermined in the revised pedigree (Fig 1).

### Identification of candidate genes by WES analysis

Each sample that underwent WES resulted in an average of 44 million paired-end (2x100 bp) sequence reads with an average quality score of 35 and exome coverage of 43X. An average of 124,730 variants were identified per individual. A total of 1,466 heterozygous SNVs in the chromosome 10q23-q24 linked interval were present in all five affected individuals and absent in the two unaffected controls. While 1,192 of the identified variants were identified in untranslated and other non-coding regions, 153 were predicted to result in synonymous substitutions and 121 were predicted to produce missense or nonsense changes. Three of these SNVs located in two genes survived all of the filtering criteria: one rare variant, c.3156C>T (p.(Gly1052 =)) in exon 46 of *COL17A1* with an undefined minor allele frequency; and two rare variants in *DNMBP*, c.2492T>C (p.(Met831Thr)) and c.2514G>A (p.(Ser2606 =)), with minor allele frequencies of 0.0088 and 0.0096 (dbSNP) and 0.0235 and 0.0258 (ESP), respectively (Table 1). These three remaining SNVs were screened for via Sanger sequencing in affected and unaffected family members (Table 2).

**Table 2:** Sanger sequencing results of filtered candidate variants for the corneal dystrophy linked to chromosome 10q23-q24.

Gene		*COL17A1*	*DNMBP*	*DNMBP*
**Strand**		-	-	-
**Genomic Position**		g.105,797,446	g.101,667,814	g.101,667,792
**Affected Individuals**	III-3	G/A	A/G	C/T
	III-6	G/A	A/G	C/T
	III-19	G/A	A/A	C/C
	IV-1	G/A	A/G	C/T
	IV-2	G/A	A/G	C/T
	IV-6	G/A	A/G	C/T
	IV-8	G/A	A/G	C/T
	IV-14	G/A	A/A	C/C
	IV-18	G/A	A/A	C/C
	IV-23	G/A	A/A	C/C
	IV-27	G/A	A/A	C/C
	V-2	G/A	A/G	C/T
	V-3	G/A	A/G	C/T
**Unaffected Individuals**	IV-10	G/G	A/A	C/C
	IV-11	G/G	A/A	C/C
	IV-24	G/G	A/A	C/C
	IV-25	G/G	A/A	C/C
	V-9	G/G	A/A	C/C
	V-11	G/G	A/A	C/C
	VI-3	G/G	A/A	C/C
**Unknown Affected Status**	V-5	G/G	A/A	C/C
	V-22	G/G	A/A	C/C

### Screening of candidate genes in additional affected and unaffected individuals

DNA samples from 13 affected family members (including the five in which WES was performed), seven unaffected family members and two family members of undetermined affected status were screened for the c.3156C>T variant in *COL17A1* and the c.2492T>C and c.2514G>A variants in *DNMBP* using Sanger sequencing (Table 2). The *COL17A1* variant was identified in all 13 of the affected family members, none of the seven unaffected family members, and in neither of the two individuals of undetermined affected status. While both *DNMBP* variants were identified in eight of the 13 affected family members, neither was present in the other five affected family members, in the seven unaffected family members or the two individuals of undetermined affected status.

### *In silico* protein analysis

SIFT and Panther predict the missense variant in *DNMBP* coding for a p.(Met831Thr) substitution to be tolerated. Polyphen-2 predicts this variant to be possibly damaging, but only with a score of 0.485 out of 1. Both NetGene2 and MutPred Splice predict that the synonymous c.2514G>A (p.(Ser2606 =)) variant in *DNMBP* does not give rise to a splice site variant.

## Discussion

Given that COL17A1 protein is a component of hemidesmosomes that are responsible for anchoring corneal epithelial cells to the epithelial basement membrane, Sullivan and colleagues identified *COL17A1* as a positional and functional candidate gene for the cause of chromosome 10q23-q24 linked corneal dystrophy.[12] While screening of the 56 exons and exon/intron junctions of *COL17A1* did not identify any presumed pathogenic mutations, one novel coding region variant was identified in the affected individual and absent in the unaffected individual that was screened. However, as this c.3156C>T variant was predicted to encode a synonymous amino acid substitution, the investigators concluded that it “cannot be considered disease-specific.”[12]

Instead of utilizing a candidate gene approach to identifying the genetic basis of chromosome 10q23-q24 linked corneal dystrophy, we used a WES approach to identify the genetic basis of this dominantly inherited disorder. As Sanger sequencing of the positional candidate genes had failed to reveal a potentially pathogenic variant, the accuracy of the boundaries determined by the previous linkage analysis were questioned.[12] We have previously encountered such an error in our search for the genetic basis of another corneal dystrophy, Schnyder corneal dystrophy. After we were unable to identify a pathogenic mutation in any of the genes in the linked interval for Schnyder corneal dystrophy, Dr. Weiss and colleagues reexamined the linkage data and identified an error in the assignment of the candidate locus due to misclassification of the affected status in one pedigree. Exclusion of this family and reanalysis resulted in the expansion of the candidate interval and the identification of pathogenic mutations in the *UBIAD1* gene, located outside of the original interval.[20, 21] Therefore, WES was employed to identify the genetic basis of chromosome 10q23-q24 linked corneal dystrophy, with the initial filtering performed for variants identified in the previously linked interval. We identified three sequence variants that met all of the filtering criteria that we employed: a missense and a synonymous variant in *DNMBP*, and a synonymous variant in *COL17A1*. However, screening of an additional 15 affected and unaffected family members demonstrated that only c.3156C>T variant in *COL17A1* segregated with the affected phenotype. This, in addition to *in silico* analysis predicting that both *DNMBP* variants are most likely tolerated and the fact that both *DNMBP* variants are identified in 2–3% of the population based on the minor allele frequency from ESP (significantly higher that the population prevalence of recurrent corneal erosions), indicates that neither is related to the pathogenesis of chromosome 10q23-q24 linked corneal dystrophy.

The synonymous c.3156C>T (p.(Gly1052 =)) variant in *COL17A1* has been determined to be pathogenic by Jonsson and colleagues.[11] After using WES to identify a missense mutation in *COL17A1* that segregated with the affected phenotype in a large family with epithelial recurrent erosion dystrophy (ERED), they reexamined the potential role of *COL17A1* in chromosome 10q23-q24 linked corneal dystrophy. Specifically, they examined whether the c.3156C>T variant could be disease-causing by altering splicing. The authors noted that three different splice site prediction programs predicted that this variant created a cryptic splice donor site. Additionally, they demonstrated using an *in vitro* splice assay that the c.3156C>T variant produced an aberrantly spliced transcript that corresponded to the introduction of a splice donor site 54 nucleotides 5’ of the wild-type exon 46 donor site, as predicted by the splice site prediction programs.[11]

Additional evidence of the pathogenic role of the c.3156C> T variant in *COL17A1* in ERED has been provided by Oliver and colleagues.[13, 14] After a genome-wide association study in a New Zealand family with a corneal dystrophy associated with early onset of recurrent corneal erosions demonstrated a signal on chromosome 10, exome sequencing identified candidate variants in three genes, including *COL17A1*. Screening of *COL17A1* in this family and in two other families with a similar phenotype confirmed segregation of the c.3156C>T variant with the affected phenotype in each family.

The vast majority of previously identified mutations associated with corneal dystrophies have been non-synonymous and within the coding regions of their associated genes.[22] To the best of our knowledge, pathogenic splice site mutations have been previously reported in only two other dystrophies: posterior polymorphous corneal dystrophy 3 and congenital hereditary endothelial dystrophy.[23, 24] The c.3156C>T variant in exon 46 of *COL17A1* described herein is only the third instance in which a mutation affecting a splice site has been associated with a corneal dystrophy and the first in which a cryptic splice site is directly introduced. While the cell-based splice site assays performed by Jonsson et al. likely represent what is occurring *in vivo*, analysis of the COL17A1 protein in tissue or cells obtained from the affected family members would validate the cell based experimental results.[11]

## Conclusions

Given the similar phenotype and clinical course in the family that we report and those recently reported by Jonsson and colleagues and Oliver and colleagues, and the fact that each has been associated with the same variant in *COL17A1*, we propose that chromosome 10q23-q24 linked corneal dystrophy be classified as ERED.[11, 14] The current International Committee for Classification of Corneal Dystrophies (IC3D) classification of the corneal dystrophies has grouped three dystrophies with erosions developing in the first decade of life followed by the development of subepithelial fibrosis under the heading of ERED: Franceschetti corneal dystrophy (FRCD), Dystrophia Smolandiensis (DS) and Dystrophia Helsinglandica (DH).[22] We join Jonsson and colleagues and Oliver and colleagues in encouraging the screening of *COL17A1* in families affected with FRCD, DS and DH to determine if a common genetic basis exists for each.
